# The influence of diet on anti-cancer immune responsiveness

**DOI:** 10.1186/s12967-018-1448-0

**Published:** 2018-03-20

**Authors:** Laura Soldati, Laura Di Renzo, Emilio Jirillo, Paolo A. Ascierto, Francesco M. Marincola, Antonino De Lorenzo

**Affiliations:** 1 Department of Health Sciences, Università degli Studi di Milano, Via A di Rudinì 8, 20124 Milan, Italy; 2 Section of Clinical Nutrition and Nutrigenomics, Department of Biomedicine and Prevention, University of Rome Tor Vergata, Via Montpellier 1, 00133 Rome, Italy; 3 Department of Basic Medical Sciences, Neuroscience and Sensory Organs, University of Bari, 70124 Bari, Italy; 4 Melanoma, Cancer Immunotherapy and Development Therapeutics Unit, Istituto Nazionale Tumori IRCCS Fondazione “G. Pascale”, Via Mariano Semmola snc, 80131 Naples, Italy; 5 AbbVie Corporation, Redwood City, CA USA

**Keywords:** Immunotherapy, Healthy diet, Cancer, Microbiome, Functional supplements

## Abstract

Immunotherapy has matured into standard treatment for several cancers, but much remains to be done to extend the reach of its effectiveness particularly to cancers that are resistant within each indication. This review proposes that nutrition can affect and potentially enhance the immune response against cancer. The general mechanisms that link nutritional principles to immune function and may influence the effectiveness of anticancer immunotherapy are examined. This represents also the premise for a research project aimed at identifying the best diet for immunotherapy enhancement against tumours (D.I.E.T project). Particular attention is turned to the gut microbiota and the impact of its composition on the immune system. Also, the dietary patterns effecting immune function are discussed including the value of adhering to a healthy diets such as the Mediterranean, Veg, Japanese, or a Microbiota-regulating diet, the very low ketogenic diet, which have been demonstrated to lower the risk of developing several cancers and reduce the mortality associated with them. Finally, supplements, as omega-3 and polyphenols, are discussed as potential approaches that could benefit healthy dietary and lifestyle habits in the context of immunotherapy.

## Immunotherapy: today’s status and future outlook

Check point inhibitor therapy has brought a paradigm shift in the treatment of advanced cancer by introducing immunotherapy as a recognized first and second line modality. The major benefit is the long-term effectiveness, that can be observed with immunotherapy agents compared to other standard therapies such as chemotherapy or pathway inhibitors. However, only a subset of cancers within each indication responds to this treatment leaving the majority of patients unaffected by this potentially curative modality [1].

We have recently shown, that human cancers evolve following a strict selection bottleneck according to a degenerate process based on genetic instability and leading to a trial and error enhancement of oncogenic processes or through an orderly stepwise accumulation of relevant driver mutations. In the former case, the degenerate and chaotic process associated with enhanced immunogenicity of cancer cells, while in the latter cancer cells adapted to grow without being recognized by the host immune surveillance [2]. Immunotherapy works only in the context of the immunogenic disorderly cancers while silent tumours are resistant. Within immunogenic tumours, only a subset responds to immunotherapy and the reasons for the unpredictable outcomes remain unknown.

Three categories of factors may determine immune responsiveness in cancer: the genetic make up of the host, the somatic profile of cancer cells and the environment [3].

While most efforts to understand immune responsiveness revolve around the somatic alterations of cancer cells and their effect on the host cells within the cancer microenvironment and in the circulation, relatively little information is available about the role played by environmental factors in modulating cancer-interactions. Here, we summarized how a fundamental aspect of daily life, dietary habits, could influence cancer growth and/or responsiveness to immune modulation. Clear variations are seeing in the functions of the human immunome according to simple variables such as age, physiological or pathological status as recently discussed by Davis et al. [4].

Several conditions have been associated with variations in immune function. However, nutri-genomics (the analysis of transcriptional programs activated by nutrients) has been championed by few. There are several ways in which nutrition can affect cancer growth through systemic or local effects within the tumour microenvironment (TME). Metabolic factors like the decrease of arginine and tryptophan level, the increased metabolism of glucose with the subsequent increase of lactate levels, and the adenosine pathway, are all well known to have an impact on the immune activity inside the TME. It is also clear that the general metabolic status determining deviations from ideal body weight highly influences the immune status. In addition, particular dietary components such as vitamins may be modulated by the nutritional status and secondarily affect immune function. Moreover, nutrition may affect the gut microbiome, which in turn has drastically different effects on immune function according to its composition [5–7]. All these aspects of nutrition within the context of the immune biology of cancer will be discussed here.

## The link between immunity and nutrition

Food represents not only a source of nutrients for body growth and for the maintenance of essential functions, but also includes dietary components that behave as antigens. Especially, innate and adaptive intestinal immune cells scattered throughout the *lamina propria* or present within secondary lymphoid organs, such as Peyer’s patches and colonic lymphoid follicles, can elicit a robust response when stimulated by food antigens. In fact, it has been demonstrated that food ingestion leads to a condition of post-prandial low-grade inflammation, which is not only regional but also systemic [8]. In healthy volunteers serum levels of the inflammatory cytokine, interleukin (IL)-17, dramatically increases 1 h after the ingestion of a western-type hyper caloric breakfast [9]. This was not observed in a control group of individuals who ingested along with breakfast polyphenols containing fruit juice. These substances are characterized by a remarkable anti-inflammatory activity, which accounts for the reduction of IL-17 serum concentration.

A trial conducted in normal weight children, who followed a Mediterranean diet (MD) for 1 year compared to age matched controls, who disregarded health food recommendations, demonstrated different immune profiles [10, 11]. Quite interestingly, at the end of the trial in the former group, salivary levels of the anti-inflammatory cytokine, IL-10, increased, while levels of IL-17 decreased. In the latter group of children, who mostly ate “*junk food*”, a dramatic increase in IL-17 was observed at the expense of IL-10. In this context, IL-10 is a cytokine predominantly produced by FOXP3+ T regulatory (TREG) cells, which are induced in the intestine by several dietary components, such as vitamins (A and D), polyunsaturated fatty acids (omega-3) and polyphenols.

The homeostatic equilibrium between TREG cells (IL-10) and Th17 cells (IL-17) is broken in different clinical settings. Overall, obesity, a diet-related disease, represents a systemic inflammatory condition characterized by an excessive production of IL-17 and IL-21, which, in turn, are strong inducers of Th17 cells [12, 13]. The intense and prolonged inflammatory status in obese people is responsible for diabetes, cardiovascular events, neuro-degeneration, and, in some cases, cancer.

Immunosenescence is known as the decline of the immune system with age accounting for increased frequency of infectious, autoimmune and neoplastic diseases in elderly [14]. Hypo-nutrition in aging aggravates the already impaired immunity, since aged people are frequently malnourished in relation to poor socio-economic conditions, mental illnesses and tooth loss [15]. Importantly, lack of proteins and oligo-elements in elderly determines severe immune deficit, which can be fatal. For instance, zinc deficit in elderly is very common, but undiagnosed, thus leading to T cell malfunction and increased frequency of respiratory infections and poor responses to vaccination [16]. Consequently, zinc supplementation in the elderly with zinc deficiency is very effective for the treatment of chronic diseases [17]. Among other natural products, evidence suggests that administration of red grape polyphenols to aged people restores impaired T cell functions, thus increasing protection against winter infections [18]. Moreover, prebiotics, probiotics and symbiotics have been shown to restore innate and adaptive immunity in elderly, also correcting alterations of intestinal microbiota which, under normal conditions, contributes to immune homeostasis, balancing the equilibrium between TREG cells and Th17 cells [19]. The immunomodulation exerted by natural products in elderly is illustrated in Table 1.

**Table 1:** Correction of immune dysfunctions with natural substances

Category name	Active compound	Effect or molecular target	Ref.
Prebiotics (fruit and vegetables)	Vitamins A, B1, B2, B6, B3, B12, D, E (tocopherols: α, β, γ, δ-tocopherol family (α T, β T, γ T, δ T) and α, β, γ, δ-tocotrienol (α TE, β TE, γ TE, δ TE)); MUFA, PUFA (ω-9, ω-6); iron and zinc; phytosterols; inuline; fiber	↓Bcl-2, ↑BAX, ↓NF-kB, ↓Cyclin D1, ↓MMP-9, ↓iNOS, ↑Caspase, ↑GPX1, ↓IRAK1, ↓IL-1, ↓CAT, ↓CCL5, ↓DUOX2, ↑SOD1, ↓COX2, ↓TNF-α, ↓IL1, ↓IL6, ↓IL8	[17, 18, 45, 149–162]
Probiotics	*Bacteroides, Clostridium, Faecalibacterium, Eubacterium, Peptidococcus, Peptidostreptococcus and Bifidobacterium*	Restoration of innate and adaptative immunity; correction of the altered intestinal microbiota; T cell differentiation toward regulatory T (Treg) cells and Th2 phenotypes; anti-inflammatory activity; stimulation of the GALT, MLNs, ILFs, TLRs, expression of α- and β-defensins, cathericidin LL-37, lectins, and other antimicrobial proteins	[20, 88, 91, 101–110, 114, 119–133]
Postbiotics	Short chain fatty acids, p40 molecule, becteriocin, Lactocepin secreted by *L. paracasei, Lactobacillus plantarum,* S-layer protein A and polysaccharide A produced by *Bacteroides fragilis*	Improved epithelial barrier function, inactive IP-10, increased production of mucins by the goblet cells, decreased inflammatory process, down-regulation of pro-inflammatory cytokine production by intestinal epithelial cells	[125, 126, 134]
Poliphenols	Resveratrol, pterostilbene, and piceatannol	↓Survivin, ↓cyclin D1, ↓cyclin E, ↑p53, ↓Bcl-2, ↑BAX, ↑Caspase, ↓Bcl-XL, ↓CIAP, ↓Egr-1, ↓PKC, ↓PKD, ↓IL-6, ↓VEGF, ↓IL-1, ↓IL-8, ↓CYP1A1, ↓5-LOX, ↑HO-1, ↑Nrf2, ↓COX2, ↑SIRT2, ↓CCL5, ↓TNF-α ↓IL-1β, ↓NF-kB, ↑IL10, ↓IL-1β, ↓IL-1β, ↑IL10	[20, 171–181, 184–188, 195–201]
	Hydroxytirosol	↓CCL5, ↓UCP2, ↓Bcl-2, ↓DUOX2, ↓IRAK1, CAT, ↓NF-kB, ↑SOD1	[163, 164]

B cell lymphoma 2 gene (Bcl-2), nuclear factor kappa B (NF-kB), matrix metalloprotease (MMP), inducible nitric oxide synthase (iNOS), glutathione peroxidase 1 (GPX1), copper chaperone for superoxide dismutase (CCL5), superoxide DISMUTASE (SOD1), interleukin (IL), gut-associated lymphoid tissues (GALT), smaller Peyer’s patches and mesenteric lymphonodes (MLNs), isolated lymphoid follicles (ILFs), Toll-like receptors (TLRs), Bcl-2-associated X (BAX), B-cell lymphoma-extra large (Bcl-XL), early growth response protein 1 (EGR1), protein kinase C (PKC), protein kinase D (PKD), vascular endothelial growth factor (VEGF), lipoxygenase (LOX), NF-E2-related factor (Nrf), cyclooxygenase-2 (COX-2), sirtuin (SIRT), tumor necrosis factor alpha (TNF-α), uncoupling protein 2 (UCP2), dual oxidase e gene (DUOX2), interleukin-1 receptor-associated kinase 2 gene (IRAK1), catalase (CAT), C-X-C motif chemokine ligand 12 (CXCL1/2)

Immune cells originating from the circulation including monocytes represent a significant component of the tumour microenvironment [20]. Monocytes differentiate into tumour-associated macrophages (TAMs), whose density positively correlates with tumour progression [20]. In highly immunogenic tumours, T cytotoxic (Tc) cells are also present, thus leading to tumour destruction or at least limitation of cancer growth. However, tumour cells and TAMs produce suppressive cytokines [for example, IL-10 and transforming growth factor (TGF)-beta], which, in turn, dampen T cell-mediated cytotoxicity [21]. Furthermore, TAMs express PD-1 ligand, which binds to PD-1 inhibiting Tc cell function [22]. Importantly, TAMs secrete the chemokines CCL17 and CCL22, which attract TREG cells, and Th2 cells to tumour site, thus down-regulating Th1 cell function [20]. It is well known that Th1-related cytokines, such as IL-2 and interferon (IFN)-gamma, allow Tc and natural killer (NK) cells to proliferate, while enhancing their function, respectively. Then, the integrity of the Th1 function is essential for Tc and NK cell-mediated tumour destruction to occur.

In a recent review, Mattner and Wirtz [23] pointed out the ambiguous role of innate lymphoid cells (ILCs) in the tumour development. Th1-type ILCs (ILC1) producing IFN-gamma and Tumour Necrosis Factor (TNF)-alpha play a predominant anti-carcinogenic activity. Th2-type ILCs (ILC2) are both pro-tumorigenic (inhibition of Th1 cells) and anti-tumorigenic (attraction of eosinophils which are cytotoxic to tumour cells). Also the role of Th17-type ILCs (ILC3) is ambiguous, since production of IL-17 and IL-22 favour tumour growth on one side, while, they may also interact with tumour cells via natural cytotoxic receptors or by forming tertiary lymphoid structure that results in cancer cell elimination. It is worth mentioning that the IL-17/IL-22 innate axis in the gut can be modulated by both polyphenols and probiotics, suggesting the potential of dietary manipulation in different clinical settings [24]. However, the exact role of ILCs in the context of cancer has to be better clarified.

The cellular composition of the tumour microenvironment leads to a status of chronic non-resolving inflammation. In fact, TAMs, as well as cancer cells, produce an array of pro-inflammatory cytokines, such as IL-1 beta, TNF-alpha and IL-6 via activation of the transcription factors NF-kB and STAT 3 [25]. In addition, release of reactive oxygen and nitrogen species (ROS, RNS) accounts for epigenetic modifications, arrest of DNA repair mechanisms and DNA mutations, which favour cancer proliferation [26]. Once established, chronic inflammation leads to fibroblast recruitment contributing to the tumour microenvironment and tissue remodelling [27]. For instance, in both hepatocellular carcinoma and pancreatic cancer fibroblasts have been shown to enhance the aggressiveness and invasiveness of tumour cells [28, 29]. Production of TGF-beta by TAMs leads to accumulation of M2-type macrophages that contribute to fibrosis and hypoxia [30]. Moreover, macrophages produce matrix metalloproteinases, which are enzymes able to degrade the extracellular matrix, thus facilitating metastatic dissemination of cancer cells.

In general terms, TAMs exert procarcinogenic effects, either generating growth factors (Epidermal Growth Factor, Fibroblast Growth Factor and Vascular Endothelial Growth Factor) [31, 32] or releasing cytokines, such as IL-6, which exhibit anti-apoptotic activities on cancer cells [33]. It is should be emphasized, however, the immune infiltrates within the tumour microenvironment are characterized by a natural plasticity and their functional orientation can be reverted by variation in the intra-tumour homeostasis induced by various exogenous agents such as immunotherapy or environmentally related factors such as co-morbidities, diet and microbiota [34, 35].

In conclusion, it is likely that an appropriate dietary regimen can maintain the equilibrium between the inflammatory pathway (triggered by Th17 cells) and the anti-inflammatory cascade of events mainly based on TREG activity. Thus, a nutritional intervention in patients with cancer, should take into account a possible imbalance in the ratio between Th17 and TREG cell function. Accordingly, dietary intake of bioactive principles with food or via the products derived from food with extra health benefits in addition to basic nutritional value, the so called nutraceuticals, should be evaluated in order to enhance anti-tumour immune response.

## Dietary patterns that affect immune function: Mediterranean diet, Veg diet, Japanese diet, or a Microbiota stimulating diet

It is in general appreciated that only long-term and consistent dietary pattern can benefit human health, or conversely, induce inflammation and increased oxidative stress if an unhealthy diet is followed, that leads to chronic disease [36].

The use of specific nutraceutics, discussed in the following paragraphs, should be framed in the broader context of the composite diet.

In this section, we will review some diet patterns that have been assessed for health benefits. We will give the historical background, and review their components.

The diet that affects our health starts from the pre-natal stage and accompanies us, with major fluctuations, for the rest of our lives. Here, we will focus on diet appropriate to the adult stage of life.

### Mediterranean diet

The description of the MD stems from the nutritionist Ancel Keys, who in 1945, in the wake of the US Fifth Army, landed in Southern Italy, where he observed one of the highest concentrations of centenarians in the world. He also noticed that cardiovascular diseases, widespread in the USA, were less frequent there. In particular, among the Southern Italians, the prevalence of “wellness” diseases such as hypertension and diabetes mellitus was particularly low [37, 38]. Keys, focused his attention on fat consumption suggesting that the main factor responsible for the observations was the type of diet traditionally consumed among people facing the Mediterranean Sea, low in animal fat, as opposed to the Anglo-Saxon diet. The link between serum cholesterol and coronary heart disease mortality was subsequently demonstrated by the Seven Countries Study [39, 40]. Later, the concept of MD was extended to a diet rich in fruits, vegetables, legumes, whole grains, fish and olive oil as the main source of lipid, shared among people living in Spain, Greece, Southern Italy and other Countries facing the Mediterranean basin [41].

A meta-analysis of twelve primary prevention studies, including a total of over a million and a half individuals followed for a period of time varying from 3 to 18 years, found a significant reduction in the risk of overall mortality and mortality due to cardiovascular disease. In addition, it was observed a reduced incidence and mortality of cancer, Parkinson’s and Alzheimer’s disease, in association with the adherence to a MD [42]. In 2010, the United Nations Educational, Scientific and Cultural Organization (UNESCO) recognized the MD as an “*Intangible Cultural Heritage of Humanity*”.

Recently, Dehghan et al. [43] published in the Prospective Urban Rural Epidemiology (PURE) study that high carbohydrate intake is associated with an increased risk of overall mortality, but not with the risk of cardiovascular disease or cardiovascular mortality. The intake of any type of fat has been associated with a lower risk of overall mortality. In the meantime, the PURE group reported in another article that the assumption of raw fruits, legumes and vegetables, as source of carbohydrates, is associated with lower mortality [44]. For greater clarity and in order to ensure a healthy diet it is important to consider the nutritional quality indexes, and the amount of bioactive food components that bear a potential preventive effect on cancer, as those in MD [45]. The healthy MD is made of a proper combination of quality foods, evaluated both on the basis of the macro and micronutrient content, but also on the absence of contaminating substances such as pesticides, fertilizers and endocrine disrupters, which can alter the intestinal microbiota [10, 46, 47].

According to current understanding, the key factors against immune-mediated inflammatory responses, such as those occurring in cancer, as well as their potential clinical application, are on one side low cholesterol levels and on the other high levels of antioxidants contained in fruits and vegetables and mono unsaturated fatty acid (MUFA) present in fish, nuts and olive oil.

Moreover, nutritional supplementation with arginine, omega-3 fatty acids and nucleotides results in a marked improvement of immune functions in cancer patients undergoing surgery and a reduction in infectious complications, hospital stay and co-morbidities [48].

New insights into the effects of MD on incidence and mortality of different types of cancer have come from a recent systematic review and meta-analysis that analysed 56 observational studies including 1,784,404 subjects [49]. The results confirmed an inverse association between the adherence to MD and overall cancer-related mortality with risk of developing several types of cancer including: breast, colorectal, gastric, prostate, liver, head and neck, pancreatic and lung cancer.

Among components of the MD, olive oil has been the subject of several epidemiological studies suggesting its protective role in cancer. Associations between increased consumption of olive oil and decreased risk of developing breast [50–53] and colorectal cancer have been observed [54–56]. The main protective effects of olive oil consumption are attributable to the presence of monounsatured fatty acids (MUFA) and phenolic compound, including simple phenols, aldehydic secoiridoids, flavonoids and lignans, although at present there is no scientific evidence determining the role played on the immune system by antioxidant or MUFA components. Oleic acid is the prevalent fatty acid, linoleic and palmitic acids are also present although in minor quantity, while antioxidants include, among others, phenols, lignans and flavonoids [57, 58]. A systematic review and meta-analysis of 13,800 cancer patients and 23,340 controls in 19 observational studies found an inverse relationship between olive oil consumption and the prevalence of breast of digestive system cancers [59]. Contradictory results were observed between intake of olive oil or its components and prevalence or mortality for prostate cancer [60–63].

### Vegetarian diet

The vegetarian diet includes various dietary patterns that have as common basis the abstinence from the meat and fish. The choice of vegetarianism in ancient times depended mostly on religious choices and it was first discussed in the cultures of ancient Greece and India [64]. In Western Countries, where meat and poultry are the base of protein intake, it is becoming increasingly popular in recent times, both for ethical reasons, against intensive farming and the pollution caused by large animals’ flatulence, and health reasons related to the carcinogenicity of red and processed meat [65]. From a theoretical point of view, a diet rich in antioxidants, fibres, monounsaturated and polyunsaturated fatty acids, should decrease cancer incidence and mortality. However, scientific evidence about the anti-cancer effect of vegetarianism remains scarce compared with the data available for MD and the results are complicated to explain.

A meta-analysis of nine studies conducted on 686,629 individuals with breast (n = 3441), colorectal (n = 4062) or prostate (n = 1932) cancer did not find any association between vegetarian compared to a non-vegetarian diet. Instead, an association was found between colorectal cancer and a semi-vegetarian diet, defined as a low consumption of meat (more than once per month but less than once per week) and also with a pesco-vegetarian diet, defined as consumption of fish more than once per month [66]. From these results, it appears that there is a high heterogeneity among dietary patterns defined as vegetarianism, which may confuses the analysis. Among them, some are considered healthy because they come close to the guidelines focusing on reducing non-communicable diseases [67–71] while others deviate from them. Strict vegans, who exclude from their diet all animal products, including eggs, dairy and honey, undergo deficiencies of vitamin B12, zinc, iron and n-3 poly unsaturated fatty acids (PUFA) [72], while controversial is the deficiency of vitamin D, which depends not only from the intake mainly from fish and seafood, but also from exposure to sunlight and skin tone [73, 74]. In this and other type of vegetarianism, including raw veganism and fruitarianism, supplements of these elements are necessary to avoid serious health problems.

Despite these limitations, a systematic review and meta-analysis including 86 cross-sectional and ten cohort prospective studies about vegetarian and vegan diets, found a significant association with incidence of cancer (− 8%). However, the number of studies taken in account was limited: 2 cross-sectional and 3 prospective studies for a total of 38,053 patients. Moreover, no significant reduction was observed for breast, colorectal, prostate and lung cancer in comparison to omnivores [75]. These most recent results, cannot, be considered definitive, both for the limited number of studies and subjects, and because do not take into account the duration of the vegetarian or vegan diet, which is undoubtedly an important element. Moreover, also individual defined as omnivores include great variability in type, frequency and amount of meat consumed.

### Japanese diet

Japanese people have the greatest life expectancy and their diet is considered one of the healthiest in the world, low in cholesterol and in caloric intake [76]. But the strengths of this diet may depend on other components. Characteristic among Japanese diet is the wide consumption of green tea, rich in flavonoids, which are phytochemicals with antioxidant and anticancer properties [77]. Moreover, high consumption of vegetables, and among them miso soup, containing wakame (Undaria pinnatifida), a healthy sea vegetable, rich in fucoxanthin, a carotenoid has great antioxidant and anticancer activity [78]. The main source of proteins is fish, especially salmon and tuna fish, sources of also n-3 PUFA. These fatty acids seem to play a critical role in affecting the incidence and growth of colorectal cancer [79], breast cancer treatment efficacy [80], and prevention of prostate cancer [81]. Another common protein source consists of the high intake of soybean products such as tofu. A large body of literature demonstrated anticancer effects of soy and its components: proteins, isoflavones and saponins in in vitro and epidemiological studies [82–85]. In conclusion, similar for the studies in western populations, in which the quality of diet is linked to a lower mortality for chronic diseases including cancer, adherence to Japanese diet is associated to similar outcomes [76].

### Very low calorie and ketogenic diets

Diets restricted in calories are recognized as a sound therapeutic strategy to reduce the risk of chronic diseases, including cancer, and increase life expectancy [86]. Aside caloric restriction, low protein consumption can impair tumour genesis and inflammation [87]. The effect of short-term starvation is related to the decrease of serum levels of glucose and insulin growth factor (IGF), which exerts a potent tumorigenic effect on a variety of cancer cells by promoting proliferation and inhibiting apoptosis [88].

Lately, several studies have demonstrated positive therapeutic effects of very-low-carbohydrate ketogenic diets (VLCKD) on different diseases [89]. Some findings suggested that VLCKD could delay cancer progression due its composition [89, 90]. Ketogenic diets are poor in carbohydrates (usually less than 50 g/day) and, consequently, more abundant in lipids and proteins. Under this condition, the human body makes use of other mechanisms to generate energy, producing ketone bodies as it occurs in food deprivation. In the last phase of food deprivation and ketogenic diets, glucose become scarce and fat-derived ketone bodies become the most prevalent source of energy, promoting a decrease of reactive oxygen species production and cell growth/proliferation [91, 92].

High glucose blood levels can increase cancer risk since glucose is the energy source for human cell proliferation, including cancerous cells [92]. Reduced glucose and insulin/IGF-1 concentrations are capable to sensitize tumor cells and improve resistance of normal cells [93]. Responsible for insulin signaling, Akt is known to induce resistance to apoptosis, changes in the cancer cells metabolism, reduction of beta-oxidation and increased synthesis of lipid in the cytosol [94].

Therefore, since carbohydrates are well-known to increase either serum glucose and insulin, a personalized VLCKD, composed in its majority by fat rather than protein, could play an important role in the treatment of oncologic patients [95, 96], also increasing normal cells protection against chemotherapy, as already observed in fasting cycles retarding growth of tumors and sensitizing a range of cancer cell types to chemotherapy [88].

### Microbiota influence on diet

In the course of evolution, several microbial ecosystems developed and created a symbiotic mutualism between host and microbes [97–99].

In humans, there are almost 3 × 10^13^ eukaryotic cells and 3.9 × 10^13^ microorganisms [100], and the microbiota exhibits considerable intra- and inter-personal variations, colonizing different habitats as oral cavity, gut, vagina, respiratory tract and skin. Moreover, bacterial genes encompass together more than 100 times the number of genes in the human genome [101, 102].

An example of symbiotic proficiency is observable in the human lower gastrointestinal tract that contains approximately 1 kg of bacteria, with a total genome (microbiome) 100 times that of the host [103] representing the largest source of non-self-antigens for the human organism [104].

In recent years, it has become clear that the gut microbiota plays an important, if not crucial, role in human’s physiology and in the development of chronic diseases, including cancer, in particular in the colorectal carcinoma [105], due to its ability to stimulate immunity as an endocrine organ, able to regulate inflammatory, metabolic, and infectious diseases [101, 102, 106].

It is now clear that the influence of the microbiota on cancer development is dependent on the maintenance of chronic inflammation or upon direct effects on immune cells [107]. In fact, observing a diet that nurtures a healthy gut microbiota is critical to human healthy and macronutrients, fibres and some micronutrients have an impact on it [108].

*Firmicutes, Bacteroidetes, Actinobacteria, Proteobacteria* and *Verrucomicrobia* represent the major phyla harbouring our intestine. Most predominant genera are *Bacteroides, Clostridium, Faecalibacterium, Eubacterium, Ruminococcus, Peptidococcus, Peptidostreptococcus* and *Bifidobacterium* [109, 110], and it is possible to categorized 3 enterotypes, Bacteroides, Prevotella and Ruminococcus on the basis of the microbiota profile [111–115]. Furthermore, gut microbiota differs in males and females, due to the influence played by androgens [116].

**Fig. 1:**
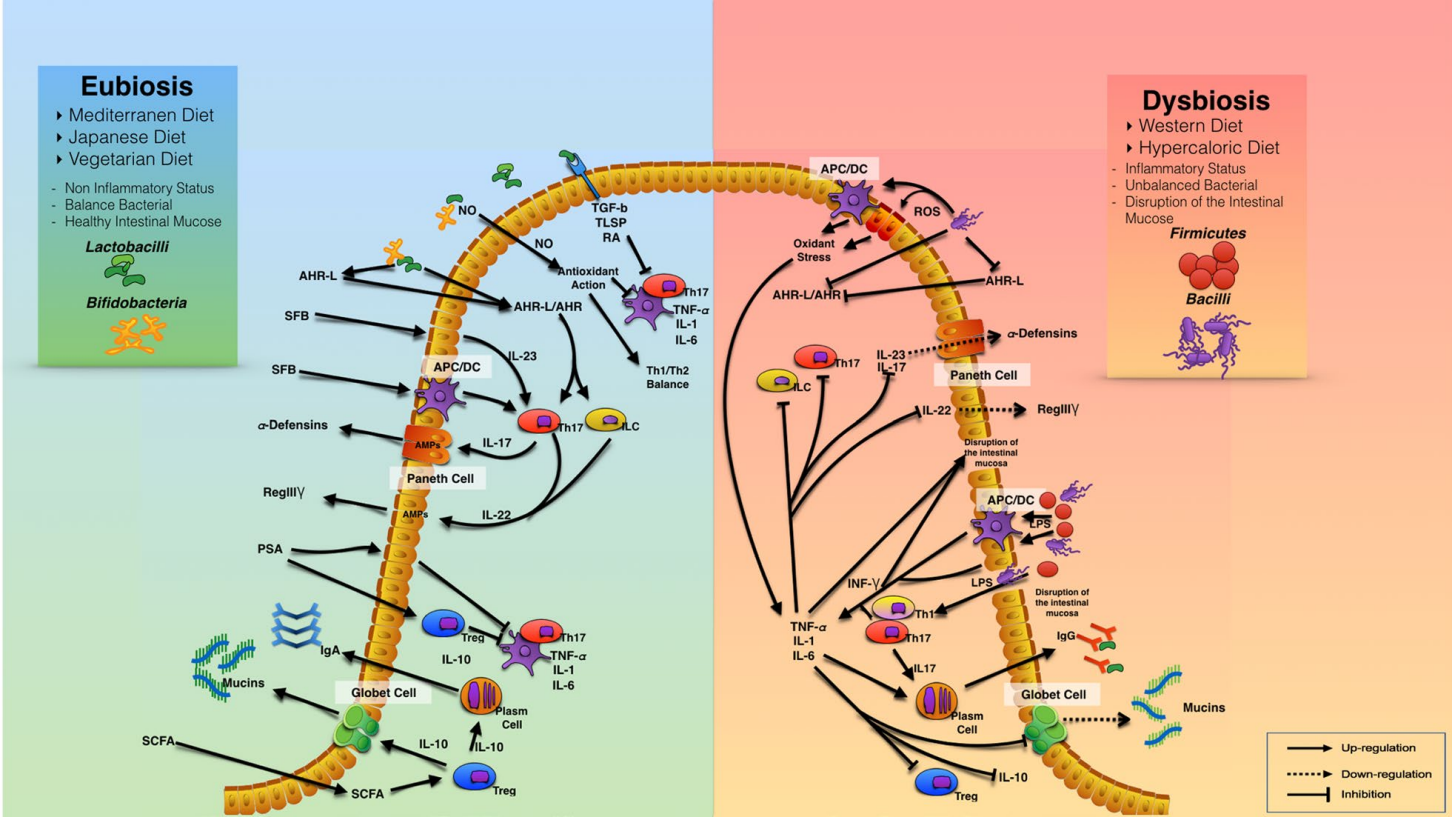
The role of dietary patterns on microbiota composition and immune system function. Dysbiosis induces cytokines production and NF-kB-mediated local inflammation, promoting tumorigenesis. In the left part of the figure are shown the main mechanisms of interaction between a healthy diet, a balanced gut bacterial flora and the immune system. In the right part are shown alterations of the immune system, intestinal barrier and pathological pathways due to an unhealthy diet and unbalanced gut microbiota

The influence of diet on the microbiota has been extensively studied, both by epidemiological and interventional studies that demonstrated that a switch in diet, from vegetarian to carnivore, results in a change in the composition of the microbiome just after 24 h [117]. Figure 1 shows the impact of microbiota on the function of the mucosal immune system.

In general, the western diet, that is high in animal protein and saturated fatty acids and low in fibre, increases the abundance of bile-tolerant microorganisms and leads to a decrease of beneficial bacteria, as *Bifidobacteria* and *Eubacteria* [118], while potentially unhealthy bacteria, as *Bacteroides* and *Clostridia,* increase. High animal protein intake, that is often high in fat, lowers the number of *Bifidobacteria* favoring *Bacteroides* and *Clostridia* compared with people who do not eat meat [119]. A high-saturated fatty acid diet increases anaerobic Enterotypes and *Bacteroides* [114, 119].

Symbiotic bacteria, through their metabolic function, are able to release essential nutrients, metabolizing indigestible compounds [120]. People consuming polyunsaturated fats possess Ruminococcus in their gut, whereas those consuming high carbohydrate and simple sugars are colonized predominantly by Bacteroides. In the microbiota of obese and overweight people, a reduction of Bifidobacterium was observed, with an increase of Firmicutes (*Roseburia* spp., *Eubacterium rectale*, and *Ruminococcus bromii*), and *Proteobacteria*, that metabolize dietary plant polysaccharides with a consequent gain in energy availability [121, 122]. Conversely, vegan and vegetarian diets, rich in fermentable nutrients, induce a microbial flora rich in healthy species [123–125].

## The impact of microbiota composition on the immune system

As reported by Stitaya Sirisinha, “*our health and probably also our behaviour and mood depend not only on what we eat or what we do (lifestyle behaviour), but also on what we host*” [126].

Researchers focused attention on the relationship between gut microbiota and brain development and function, discovering a bidirectional communication between them, defined a microbiota-gut-brain axis. Recent data highlighted the efficacy of probiotics for prevention and/or treatment of certain eating behaviour disorders and anxiety [127, 128]. Moreover, microbiota diversity plays a crucial role in the maturation and development and functions of both innate and adaptive immune system, [129, 130]. It seems, that the human genome cannot encode all the information necessary to guarantee our health and that this deficiency is overcome by molecules secreted by the intestinal microbiome (the collective genomes of the microbiota) [129, 131].

The interaction between commensal microbes and immune system is bidirectional, and intestinal bacterial species can impact the function of one another [132] (see Fig. 1).

Microbiota can act on several cell types, as intraepithelial lymphocytes, predominantly dominated by T cells of the γδ phenotype, and dendritic cells, and stimulate the gut-associated lymphoid tissues, smaller Peyer’s patches and mesenteric lymphonodes, isolated lymphoid follicles, Toll-like receptors, expression of α- and β-defensins, cathericidin LL-37, and other antimicrobial proteins. The ultrastructure of the gut is related to microbiota, and intestinal epithelial cells (IECs) can secrete and respond to various cytokines and chemokines and express molecules interacting with lymphocytes [133]. On the other hand, IECs secrete mucins and AMPs that limit microbial interaction with epithelial cells. IECs express CD1d, an MHC-like molecule that presents glycolipids to NK T cells and after the activation of STAT3 produce the anti-inflammatory IL-10 [134].

Gram-negative commensal organism *Bacteroides thetaiotaomicron*, but not the Gram-positive microbe, *Bifidobacterium longum*, stimulates IECs to produce antimicrobial peptide (AMP), α- and β-defensins, C-type lectin (e.g., regenerating islet-derived protein, REGIIIγ), cathelicidins, lectins and a number of hydrolytic enzymes [135], and to increase the expression of matrix metalloproteinase (MMP) needed for cleavage of inactive pro-defensins to active defensins [136]. Moreover, Sonnenburg et al. highlighted that *B. longum* can increase the diversity of polysaccharides that can be degraded by *B. thetaiotaomicron* [137]. *Bacteroides thetaiotaomicron* down-regulate inflammatory response because interfere with the activation of nuclear factor kappa-light-chain-enhancer of activated B cells (NFκB), in the peroxisome proliferator-activated receptor-γ (PPARγ)-dependent pathway [138].

Some commensal bacteria can secrete mediators that exert anti-inflammatory activity, as TGF-β, thymic stromal lymphopoietin, IL-25, IL-33 and IL-10. They also endow DCs and resident macrophages (CX3CR1+) T cell differentiation toward regulatory TREG cells and Th2 phenotypes.

*Bacteroides fragilis* produced immunosuppressive polysaccharide A that can also function as a TLR2 ligand, thus promoting TREG cell differentiation [139]. Moreover, TREG cell differentiation is observed after production of TGF-β due to signal by some species of *Clostridium* in and after the recognition of G protein-coupled receptors present on T cells and IECs by the short chain fatty acids (e.g. butyrate, propionate and acetate) produced as metabolites by microbiota [140]. Tolerogenic DCs produce TGF-β and RA that stimulate the development of TREG cells [141].

There is a crosstalk among innate lymphoid cells (ILCs; ILC1, ILC2 and ILC3) located in mucosal epithelium, local immune cells and epithelial cells. ILC2s secrete interleukin as IL-4, IL-5, IL-9 and IL-13, and ILC3 s secrete predominantly IL-17 and IL-22 that activate the epithelial and goblet cells to secrete AMPs and mucins able to influence the composition of the microbiota.

Certain components of the inflammasome, such as Nod-like receptor pyrin domain 6, are selectively expressed by intestinal epithelial cells and can influence the composition of the intestinal microbiota by inducing IL-18 expression [142, 143].

As an immuno-compromised state characterized by pathobiont overgrowth leads to the loss of barrier integrity, hyperinflammation, dysplasia, and tumorigenesis, it is important to develop new strategies for the treatment of diseases associated with low-grade chronic inflammation.

*Fusobacterium nucleatum, enterotoxigenic Bacteroides fragilis, and colibactin*-*producing Escherichia coli* generate an inflammatory environment and promote tumorigenesis, such as in colorectal cancer, due to the development of the inflammasome and activation of the NF-κB pathway [144].

However, many of the anti-inflammatory food components, as dietary fibers, omega-3 fatty acid and some vitamins, tryptophan and tryptophan-derived products, and SCFAs are able to activate the production of anti-inflammatory cytokines (IL-10 and IL-22) through binding to the arylhydrocarbon receptor and the G-protein-coupled receptors [145].

On the other hand, some Lactobacillus strains (*L. casei, L. plantarum, L. acidophilus, and L. delbrueckii* subsp. *bulgaricus*) have inhibitory effects on pathogens, due to the modulatory action of TGFβ-expressing T cells, dendritic cells and macrophages, and production of IL-10 [146, 147].

Moreover, *Bacteroides* spp., *Lactococcus lactis*, *Bifidobacterium animalis* subsp. *Lactis* exert and antinflammatory activity, with the production of nitric oxide, shifting the Th1/Th2 balance, and preventing carcinogenesis, through restoration of impaired IL-12 production. They have also direct cytotoxic effects on cancer cells [148, 149].

Interaction between molecules or factors produced by the intestinal probiotics during food metabolism, such as short chain fatty acids, p40 molecule, bacteriocin, polysaccharide A, could be considered as postbiotics. Postbiotics may be able to act directly or indirectly on the metabolic processes of the host, improving epithelial barrier function. For example, it has been demonstrated that lactocepin produced and secreted by *L. paracasei,* is able to inactivated CXCL-10, a lymphocyte recruiting chemokine produced by epithelial cells. *Lactobacillus plantarum* is able to increase production of mucins by the goblet cells. More generally, protein released by probiotics, as S-layer protein A and polysaccharide A are able to decrease inflammatory process, regulating the balance between pro and anti-inflammatory cytokines by DC and T cells [150].

Finally, understanding how to best manipulate the microbiome, controlling therefore the human immune system and its dysregulation, or controlling the effects of postbiotics in the symbiotic status represents an important opportunity to develop new drugs, and combining probiotic supplements, with vaccines and cancer immunotherapies.

## Functional supplements

Dietary natural compounds, also called *phytochemicals,* can influence cancer risk and tumour behaviour, interfering in all carcinogenic steps, invasion, proangiogenic and the metastatic phase. Therefore, phytochemicals represent a valuable source of effective immune modulators for novel antitumor therapeutic strategies.

Furthermore, advances in nutrigenetics, as individual genetic “make-up” [151–154], and nutrigenomics [155], as the modulation of the whole genome expression induced by food, sustain the crosslink among nutrients–genes–cancer. Moreover, dietary phytochemicals are recognized to activate or suppress oncogenic noncoding regulatory RNAs network (miRNA), or restore normal expression level of miRNAs with tumour suppressor role [156–158].

Due to the fact that inflammation contributes significantly to the development of chronic non-communicable diseases (CNCD), including cancer [159, 160], it is of fundamental importance to select dietary phytochemicals that can modulate expression genes and miRNAs related to inflammasome pathway leading to regulate target immune systems in defined tumor microenvironments.

Inflammation results from an over-reacting immune response and is characterized by the production of different reactive oxygen/nitrogen species and pro-inflammatory mediators including lipid mediators, notably prostaglandins and leukotrienes, and cytokines like TNF-alpha and IL-6, which in turn aggravate inflammation and lead to excessive damage to host tissues [161, 162]. These activate specific patterns of gene expression that in turn act to alter the hormetic mechanisms, i.e. the biphasic dose response phenomenon, characterized by a low dose stimulation and high dose inhibition, that increase cellular stress resistance. This alteration produces tissue degeneration, loss of function of one or more organs, activating oncogene products and/or inactivating tumour-suppressor proteins [161].

Several observational studies have provided scientific evidence that diets rich in fruit, vegetables, legumes, whole grains, fish, low-fat dairy products, and hazelnut, reducing the oxidative processes and inflammation [45, 163] are associated with lower incidence of CNCD [164–166]. Moreover, dietary supplementation with antioxidants, including minerals, vitamins and phenolic compounds obtained from plants, exert health benefits, maintaining a desirable pro oxidative/anti-oxidative balance [165–168].

Omega 3, 6 and 9 fatty acid, fat-soluble bioactives with nutraceutical property (tocopherols and phytosterols), vitamins (vitamins B1, B2, B6, niacin, thiamin and α-tocopherol, the most active form of vitamin E), essential minerals (selenium, potassium, magnesium, phosphorus, manganese, iron, zinc and copper, and a low level of sodium), essential amino acids, antioxidant phenolics (caffeic acid), dietary fiber (soluble), flavonoids (as catechin, epicatechin, quercetin, procyanidins, phenolic acids (as gallic and protocatechuic acids) can be considered functional foods, that exert physiological benefits beyond basic nutritional function [45, 46, 165, 166].

Because there is a plethora of phytochemicals that appear to be protective against cancer and CNCD, and, in the meantime, there are multiple pathways that may be influenced simultaneously, we have selected some examples of nutraceuticals that act against inflammation and oxidative stress.

**Fig. 2:**
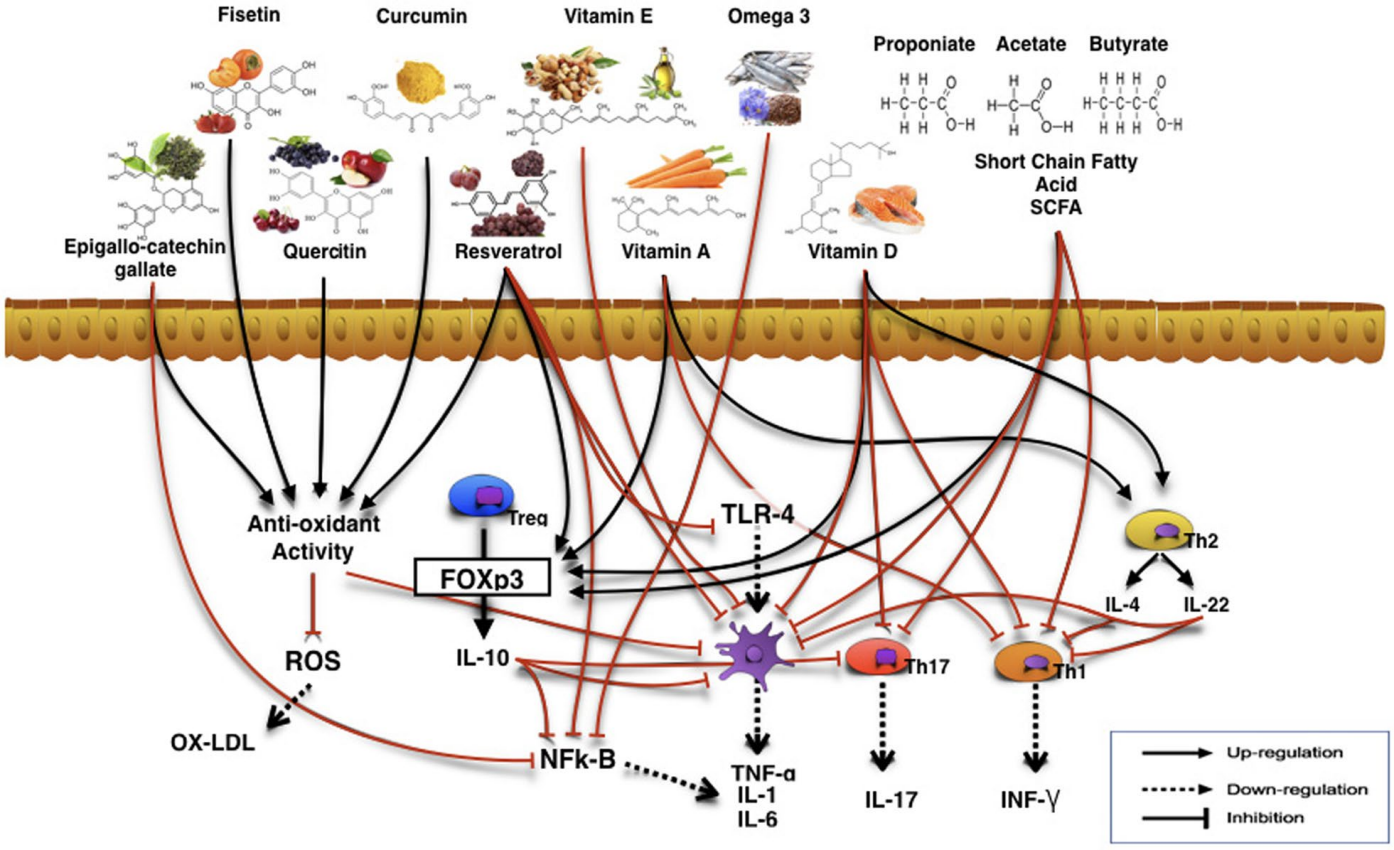
The benefits of functional nutrients on immune system and inflammation. Role of natural compounds on molecular target to correct immune system alterations, prevent and modulate the onset of cancerous disease

Figure 2 shows the dietary impact on immune system.

Vitamin E, a fat-soluble physiological antioxidant belonging to α, β, γ, δ –tocopherol family (α T, β T, γ T, δ T) and α, β, γ, δ -tocotrienol (α TE, β TE, γ TE, δ TE) [168] are potent antioxidants with lipoperoxyl radical-scavenging activities. Specific forms of vitamin E, such as γ T, δ T and tocotrienols (esp. γ TE), have anti-inflammatory and antioxidant effects by inducing superoxide dismutase, quinoneoxidoreductase, glutathione peroxidase and by inhibiting cyclooxygenase (COX)-2, signal transducer and activator of transcription-3 (STAT3), nuclear factor kappa-light-chain-enhancer of activated B cells (NF-κ B), TNF-α, cytokines as interleukin (IL-1, IL-6, IL-8), and inducible nitric oxide synthase [169–178] (see Table 1).

It has been demonstrated that the hydroxytirosol (2-(3,4-dihydroxyphenyl) ethanol, 3,4-DHPEA, HT) has positive effects on antioxidant enzymes activity, against oxidative stress, and DNA damage [179]. Oral administration of gastro-resistant capsules containing 15 mg/day of HT significantly increased antioxidant biomarkers, like thiols groups and total antioxidant status, while drastically reduced nitrite and nitrate, malondialdehyde (MDA), and peroxidation of low density lipoprotein cholesterol serum concentrations. These results were related to the significant up-regulation of superoxide dismutase-1 gene expression [180] (see Table 1).

Recently, Bhandari et al. showed that plant extract from *Allium wallichii*, rich in flavonoids, steroids, glycosides and terpenoids exhibits a good cytotoxicity towards Lymphoma, and moderate cytotoxic activity towards prostate cancer (PC3), cervical cancer (HeLa) and breast cancer (MCF-7) cell lines, and could be an important candidate to be used as an anticancer agent [181].

Epidemiological studies suggest that a higher flavonoid intake is associated with a lower cancer risk [182], affecting all three stages of carcinogenesis (initiation, promotion, and progression) by modulating the signal transduction pathways, controlling cell division and growth, apoptosis, inflammation, angiogenesis, and metastasis. Flavonoid supplementation is therefore considered a promising anticancer therapy [183].

Although food supplies with native forms of phytochemicals can achieve the maximal antitumor effect, clinical effects of these compounds can be reached at high concentrations, impossible to be retrieved from natural sources. Therefore, efforts to synthesize new derivatives are in progress [184].

Considering the complex, often synergistic, beneficial effects of mixtures of bioactive compounds present in a healthy diet, phytochemicals may be a novel adjuvant approach useful in combination with chemotherapeutics for overcoming drug resistance or tumour metastasis in therapy against cancer.

## Anti-neoplastic activities exerted by polyphenols

In the light of the aforementioned concepts, targeting of inflammatory cells or neutralization of pro-inflammatory cytokines in the context of cancer may represent a plausible innovative approach to retard or abrogate tumour growth and influence the effectiveness of anti-cancer immunotherapy. According to large population studies, there is evidence that the daily use of non-steroidal anti-inflammatory drugs, such as aspirin to prevent cardiovascular risk, significantly diminished the risk of colorectal and receptor-positive breast cancer [185]. However, the practical limit of this study is the unease of applying this therapeutic approach to healthy people in the absence of cardiovascular risk. On the other hand, use of monoclonal antibodies against pro-inflammatory cytokines (TNF-alpha and IL-6) in different cancers led to sporadic disease stabilization, thus suggesting the poor efficiency of such a therapeutic strategy [20].

With regard to nutritional interventions in cancer, the anti-oxidant and anti-inflammatory properties of polyphenols have been object of intensive investigation. Daily ingestion of polyphenols with fruits, vegetables, cereals, extra virgin olive oil, wine, tea and coffee has also prompted further studies on their anti-cancer activity.

Oolyphenols exhibit broad structural variation in their backbone as well as primary and secondary structures due to differences in methylation, glycosylation and hydroxylation, which result in diverse biological activity [186].

Anti-inflammatory activities displayed by polyphenols, especially flavonoids extracted from red wine or fermented grape marc (FGM), depend on multiple mechanisms.

Resveratrol (3,5,4′-trihydroxystilbene), a naturally occurring polyphenol produced by some plants as a self-defence agent, acts as a phytoalexin, and it is considered to have beneficial effects also on human health. Resveratrol has a wide range of healing and preventive properties, acting as a cardio-protective, neuro-protective and antitumor, antioxidant agent [187]. Furthermore, resveratrol has been shown to induce anti-oxidant enzymes (e.g. glutathione peroxidase, heme-oxygenase, superoxide dismutase) that attenuate oxidative stress [188].

Resveratrol is a potent inhibitor of cyclooxygenase-2 [189], and, therefore, a potent chemo-preventive agent against cancer (prostate, breast, brain, endometrium, rectum, pancreas, skin, lung, ovary and bladder cancer), activating pro-apoptotic signalling molecules while inhibiting anti-apoptotic molecules [190].

In particular, resveratrol is able to activate sirtuins (SIRT), a class of proteins that possesses deacetylase or monoribosyltransferase activity [191]. SIRT acts by deacytylating transcription factors, such as the tumour suppressor p53, the Forkhead Box proteins family and the transcription factors NF-kB [192, 193]. Di Renzo et al. observed, after the intake of red wine, a higher expression of SIRT2, which was negatively correlated (P < 0.001) with the expression of CCL5 [194].

Red wine polyphenols, when incubated with healthy human monocytes have been shown to interfere with the binding of lipopolysaccharides from gram-negative bacteria to Toll-Like receptor (TLR)-4, likely acting by sterical hindrance [195]. The activation of the transcription factor NF-kB was inhibited, thus leading to a dramatic decrease in pro-inflammatory cytokine production.

Moreover, resveratrol and its analogues pterostilbene (Pter; trans-3,5-dimethoxy-4′-hydroxy-stilbene), and piceatannol (Pic; trans-3,5,3′, 4′-tetrahydroxystilbene), regulate miRNAs, causing apoptosis, cell cycle arrest, growth inhibition, inhibition of cell viability, migration, and invasion in various types of cancer: 71 miRNAs are overexpressed in lung cancer cells, 46 miRNAs target TGFβ pathways in colon cancer cell, miR-125b-5p, miR-200c-3p, miR-409-3p, miR-122-5p and miR-542-3p are involved in breast cancer cells [195, 196].

Curcumin, green tea, polyphenols [epigallo-catechin-gallate (EPGC)], quercetin and resveratrol are the most effective anti-cancer compounds as they inhibit NF-kB activation [196]. The administration of FGM-derived polyphenols to mice affected by experimental colitis dramatically reduced biomarkers of inflammation such as TNF-alpha and IL-1 beta [197]. Thus, one can hypothesize that polyphenols can impede secretion of cytokines usually released in the tumour microenvironment. Finally, FGM-derived polyphenols activate in vitro peripheral healthy human Foxp3+ TREG cells inducing the production of the anti-inflammatory cytokine IL-10, and polarizing immune responses toward a tolerogenic pathway [198] (see Fig. 2).

From an immunological point of view, polyphenols from blueberry powder were very effective either in vitro or in vivo at inhibiting breast cancer cell proliferation and metastasis, down-regulating IL-6 production [199]. Similarly, curcumin could inhibit the metastatic dissemination of breast cancer, reducing the release of CXCL1/2 [200]. EGCG from green tea induced apoptosis in gamma-irradiated breast cancer cells via inhibition of NF-kB [201]. Xanthohumol, a prenylated flavonoid extracted from hops, when inoculated to nude mice bearing breast cancer xenografts, reduced macrophage infiltrates, increased apoptosis, reduced micro-vessel number and down regulated NF-kB expression [202].

Regulation of ROS by polyphenols is another potential anti-tumour mechanism. Of note, polyphenols are able to scavenge not only ROS but also RNS, peroxynitrous acid, chlorine species and hypochlorous acid by targeting NF-kB and mitogen activated protein kinase-related pathways [203]. Experimentally, Biochanin A, an isoflavonoid extracted from red clover, prevents induction of mammary gland cancer in pre-puberal rats exposed to carcinogenic agents thanks to the reduction of oxidative stress [204]. Resveratrol inhibits 17 beta-estradiol-induced carcinogenesis by up-regulating the expression of nuclear factor erythroid-related factor 2, which has anti-oxidant effects [205]. Similarly, curcumin reduced proliferation of breast cancer cells via nuclear translocation of Nrf-2 [206]. EGCG at low concentrations reduced ROS generation in response to exposure to environmental carcinogens via up regulation of NADPH-quinone oxidoreductase-1, a detoxification enzyme in phase 2 [177]. Conversely, evidence has been provided that polyphenols are also able to exert a pro-oxidant effect, which leads to anti-cancer activity. For instance, in breast cancer cells genistein, a soy isoflavone, generated a pro-oxidant effect via mobilization of copper ions with DNA damage, increase in ROS production and, ultimately, apoptosis [207]. Also, curcumin and EGCG demonstrated pro-oxidant effects in breast cancer cells by increasing ROS generation [208, 209].

Apoptosis of cancer cells is vigorously induced by polyphenols. Quercetin has been shown to induce apoptosis of MCF-7 breast cancer cells binding to the Fas/CD95 receptor via activation of caspase-6 [210]. In addition, quercetin inhibites MDMBA-231 breast cancer cells by activating caspases -3/-8/-9 [211]. Apigenin enhanced apoptosis pathway in SKBR3 breast cancer cells via inhibition of STAT3 [212]. EGCG applied to MD-MB-231 human breast cancer cells led to reduced cell growth and apoptosis related to stimulation of Bcl-2-associated X protein (BAX), cleavage of poly (ADP-ribose) polymerase protein (PARP) and reduction of Bcl-2 expression [213]. Resveratrol significantly diminished growth of estrogen-positive breast cancer cells inducing apoptosis via reduction of Bcl2/BAX ratio. Genistein induced apoptosis of MCF-7 cells blocking the activation of Insulin-like Growth Factor receptor and the phosphorylation of protein kinase B (AKT) [214, 215]. Fisetin, a flavonoid present in fruits and vegetables induced apoptosis in MCF-7 cells activating caspases -7/-8/-9, cleaving PARP, depolarizing mitochondrial membrane, increasing p53 and breaking the plasma membrane in the absence of changes in DNA or phospatidylserine [216].

Autophagy occurs in many cells of the body, even including immune cells and represents a mechanism of protection against starvation, dietary restriction oxidative stress and toxicity. The autophagy process implies catabolic lysosomal degradation, which provides an additional source of energy for the synthesis of new proteins and the maintenance of cellular homeostasis. Autophagy seems to play two main roles in tumour development [217]. Its deficiency has been shown to promote cancer growth, on the one hand. On the other hand, it attenuates necrosis and inflammation in the context of tumour, thus limiting stress-related chromosomal damage. As recently reviewed by Jin and associates [218] autophagy activates innate immune receptors as well as T and B lymphocytes for tumour destruction.

Polyphenol-induced autophagy has been considered as a mechanism of tumour cell death. Resveratrol-induced autophagy has been shown to be very effective against cancer growth. This is a type of ROS-triggered autophagy, which occurs via up-regulation of microtubule-associated protein 1 light chain 3-II [219]. Conversely, in human colorectal cancer genetic inhibition of autophagy-related proteins, such as phosphoinositide 3-kinase, lysosome-associated membrane protein 2b and Beclin1 abrogated resveratrol-dependent cell death [220]. Quercetin has been shown to induce cytoprotective autophagy in gastric cancer cells which was mediated by hypoxia-induced factor 1 alpha and AKT- mammalian target of rapamycin 1 (mTOR) pathways [221]. Modulation of the mTOR signalling occurs via inhibition of the phosphorylation level of the ribosomal S6 subunit through activation of p70S6 kinase and 4e-BP1 [222]. Genistein treatment of ovarian cancer cells led to autophagy via inhibition of AMP-phosphpdiesterase-4A4 and p62/sequestome 1 aggregates activated by extracellular signal-regulated kinases and protein kinase C inhibitors [223].

Curcumin treated cancer cells underwent autophagosome formation and cell death, which was mediated by ROS generation [224]. Similar effects have been observed in ovarian cancer cells and in oral squamous cell carcinoma where besides autophagy curcumin also induced apoptosis inactivating Bcl-2 protein and NF-kB in cancer cells [225].

## Conclusions

The current review highlights the various processes in which nutrient intake could modulate directly or indirectly the immune system and/or the growth of cancer.

Most of the discussion is based on human observation rather than experimental animal models, as the focus of this review was predominantly based on epidemiological grounds. But several experimental models not discussed here extensively substantiate the conclusions.

Yet a gap of knowledge is clear. While the potential mechanisms that may affect immune function and consequently cancer growth and responsiveness to immunotherapy agents have been discovered, very little is known about how they may affect and modulate therapies since parameters linking dietary habits to clinical outcome during immunotherapy are not routinely included.

Here we propose that in the future, detailed information about diet, nutritional status and gut microbiota should be considered in correlative studies during immunotherapy trials identifying parameters that may be relevant to outcome by studying either systemic effects of diet of circulating immune cells, or those that may affect directly the cancer microenvironment. A project is on going to identify the best diets for immunotherapy enhancement against tumours (D.I.E.T project).

## Abbreviations

AMP: antimicrobial peptide
BAX: Bcl-2-associated X protein
CCL: chemokines
CD1d: cluster of differentiation 1
CNCD: chronic non communicable disease
COX-2: cyclooxygenase-2
DCs: dendritic cells
EPGC: epigallo-catechin-gallate
FGM: fermented grape marc
HT: hydroxytirosol
IEC: intestinal epithelial cell
IFN: interferon
IGF: insulin growth factor
IL: interleukin
ILC: innate lymphoid cell
MD: Mediterranean diet
MDA: malondialdehyde
MHC: major histocompatibility complex
MiRNA: small noncoding regulatory RNA
MMP: metalloproteinase
mTOR: mammalian target of rapamycin 1
MUFA: mono unsaturated fatty acid
NFκB: nuclear factor kappa-light-chain-enhancer of activated B cells
NK: natural killer
n-3: polyunsaturated fatty acids omega-3
Nrf-2: nuclear factor erythroid-derived 2
Ox-LDL: low density lipoproteins oxidation
PARP: poly (ADP-ribose) polymerase protein
PPARγ: peroxisome proliferator-activated receptor-γ
PUFA: poly unsaturated fatty acid
PURE: Prospective Urban Rural Epidemiology
RA: retinoic acid
REGIIIγ: regenerating islet-derived protein,
ROS: reactive oxygen species
RNS: reactive oxygen species
SCFAs: short chain fatty acids
SIRT: sirtuin
STAT3: signal transducer and activator of transcription-3
Th: T helper cell
TAM: tumour associated macrophage
Tc: T cytotoxic
TGF: transforming growth factor
TLR: Toll-like receptor
TME: tumour micro environment
TNF: Tumour Necrosis Factor
TREG: regulatory T cell
VLCKD: very-low-carbohydrate ketogenic diets
